# A fluorescence-based assay for measuring polyamine biosynthesis aminopropyl transferase–mediated catalysis

**DOI:** 10.1016/j.jbc.2024.107832

**Published:** 2024-09-27

**Authors:** Pallavi Singh, Jae-Yeon Choi, Weiwei Wang, Tukiet T. Lam, Philip Lechner, Christopher D. Vanderwal, Sovitj Pou, Aaron Nilsen, Choukri Ben Mamoun

**Affiliations:** 1 Department of Internal Medicine, Section of Infectious Diseases, Yale School of Medicine, New Haven, Connecticut, USA; 2 Keck MS & Proteomics Resource, Yale School of Medicine, New Haven, Connecticut, USA; 3 Department of Molecular Biophysics and Biochemistry, Yale University, New Haven, Connecticut, USA; 4 Department of Chemistry, University of California, Irvine, California, USA; 5 Department of Pharmaceutical Sciences, University of California, Irvine, California, USA; 6 VA Healthcare System, Medical Research Service, Portland, Oregon, USA; 7 Department of Chemical Physiology and Biochemistry, Oregon Health & Science University, Portland, Oregon, USA; 8 Department of Microbial Pathogenesis, Yale School of Medicine, New Haven, Connecticut, USA; 9 Department of Pathology, Yale School of Medicine, New Haven, Connecticut, USA

**Keywords:** putrescine, spermidine, spermine, polyamines, fluorescence assay, 1,2-diacetyl benzene, aminopropyl transferases, enzyme activity, kinetics, *Plasmodium*, yeast, *Babesia*, drug discovery, inhibition, isoindole

## Abstract

Polyamines are polycationic molecules that are crucial in a wide array of cellular functions. Their biosynthesis is mediated by aminopropyl transferases (APTs), which are promising targets for antimicrobial, antineoplastic, and antineurodegenerative therapies. A major limitation in studying APT enzymes, however, is the lack of high-throughput assays to measure their activity. We have developed the first fluorescence-based assay, diacetyl benzene (DAB)-APT, for the measurement of APT activity using 1,2-DAB, which forms fluorescent conjugates with putrescine, spermidine, and spermine, with fluorescence intensity increasing with the carbon chain length. The assay has been validated using APT enzymes from *Saccharomyces cerevisiae* and *Plasmodium falciparum*, and the data further validated by mass spectrometry and TLC. Using mass spectrometry analysis, the structures of the fluorescent putrescine, spermidine, and spermine 1,2-DAB adducts were determined to be substituted 1,3-dimethyl isoindoles. The DAB-APT assay is optimized for high-throughput screening, facilitating the evaluation of large chemical libraries. Given the critical roles of APTs in infectious diseases, oncology, and neurobiology, the DAB-APT assay offers a powerful tool with broad applicability, poised to drive advancements in research and drug discovery.

Polyamines are polycationic aliphatic biogenic molecules containing carbon chains of varying lengths and different numbers of amino groups. They are found ubiquitously in all eukaryotic and prokaryotic organisms and are essential for cell growth, differentiation, and survival (1, 2). The most common polyamines include putrescine (diamine), spermidine (triamine), and spermine (tetramine) (2). The pathway for the biosynthesis of polyamines has long been considered an attractive target for the development of novel therapies for the treatment of microbial infections, cancer, and neurodegeneration (3, 4, 5, 6, 7). In various organisms, including *Plasmodium falciparum*, the main causative agent of human malaria, the polyamine biosynthesis pathway initiates with the decarboxylation of ornithine *via* ornithine decarboxylase to form putrescine (8, 9). Spermidine synthase (SPDS), a member of the aminopropyl transferase (APT) class of enzymes, then catalyzes the transfer of the propylamine group from decarboxylated SAM (dc-SAM) to putrescine to form spermidine (10). Spermidine can also accept an aminopropyl group derived from dc-SAM to form spermine, a reaction catalyzed by a second APT enzyme, spermine synthase (SPMS). In yeast, SPDS and SPMS activities are catalyzed by the Spe3 and Spe4 enzymes. Genetic studies demonstrated that disruption of *SPE3* gene results in spermidine, spermine, β-alanine, or pantothenic acid auxotrophy, whereas loss of *SPE4* results in spermine, β-alanine, or pantothenic acid auxotrophy (11). These findings highlight the importance of polyamine biosynthesis as an attractive target for the development of new antimicrobial drugs. Unlike *P. falciparum* and *Saccharomyces cerevisiae*, other lower eukaryotes, such as *Babesia* and *Eimeria* species, lack an ornithine decarboxylase enzyme and rely exclusively on the uptake of polyamines for survival (12, 13). While polyamines have been implicated in various cellular functions, data available so far suggest that one of the crucial functions of the polyamine biosynthesis pathway is to form spermidine, which serves as a precursor for the synthesis of hypusine, an uncommon but critical amino acid for the activity of the eukaryotic translation factor eIF5A (14).

Although APT enzymes involved in polyamine biosynthesis have been known for many years to be attractive targets for the development of new antimicrobials, means to inhibit their activity have relied primarily on the use of substrate analogs such as α-difluoromethylornithine (ornithine analog) and 1-aminooxy-3-aminopropane (15, 16). A search for new small molecules or natural products with specific and more potent activity has been hampered by the lack of assays that are amenable to high-throughput screening of chemical libraries. Measurement of the activity and catalytic parameters of APT enzymes has so far relied mainly on the use of radioisotope-based assays, involving radiolabeled substrates, such as [^14^C]Spermine trihydrochloride and decarboxylated S-adenosyl[methyl-^3^H]methionine, which can be incorporated into the target substrate by the APT (17, 18). A second method involved dansylation of polyamines synthesized through reactions with dansyl chloride, followed by quantitative analysis using HPLC (19). A third approach involved capillary electrophoresis with laser-induced fluorescence by derivatizing spermidine with 7-fluoro-4-nitrobenzo-2-oxa-1,3-diazole (20). Finally, SPDS activity was assessed using a specific mAb against the reaction product, 5′-methylthioadenosine (MTA), coupled with a homogeneous time-resolved fluorescence technique (21). While these four assays are sensitive, they are low-throughput and involve time-consuming procedures. Together, these data highlight the need for a new assay that could be used in a high-throughput format to screen chemical libraries.

Here, we report the development of a simple and easy-to-use assay (1,2-diacetyl benzene [DAB]-APT) for the measurement of APT enzyme activity. The assay, which can be used in 96- and 384-well formats, uses the primary amine reactive 1,2-DAB, which forms fluorescent conjugates with putrescine, spermidine, and spermine, with fluorescence intensity increasing with the length of each of these polyamines. The DAB-APT assay was evaluated and validated using the Spe3 and Spe4 enzymes of *S. cerevisiae* and the SPDS enzyme of *P. falciparum* and shown to be suitable for determining their biochemical activity, catalytic properties, and inhibition by known inhibitors, setting the stage for future chemical screens to identify new drugs with antimicrobial activity as well as potential applications in other fields such as cancer and neurodegeneration.

## Results

### Putrescine, spermidine, and spermine interact with DAB to form fluorescent adducts with increasing fluorescence intensity

**Figure 1:**
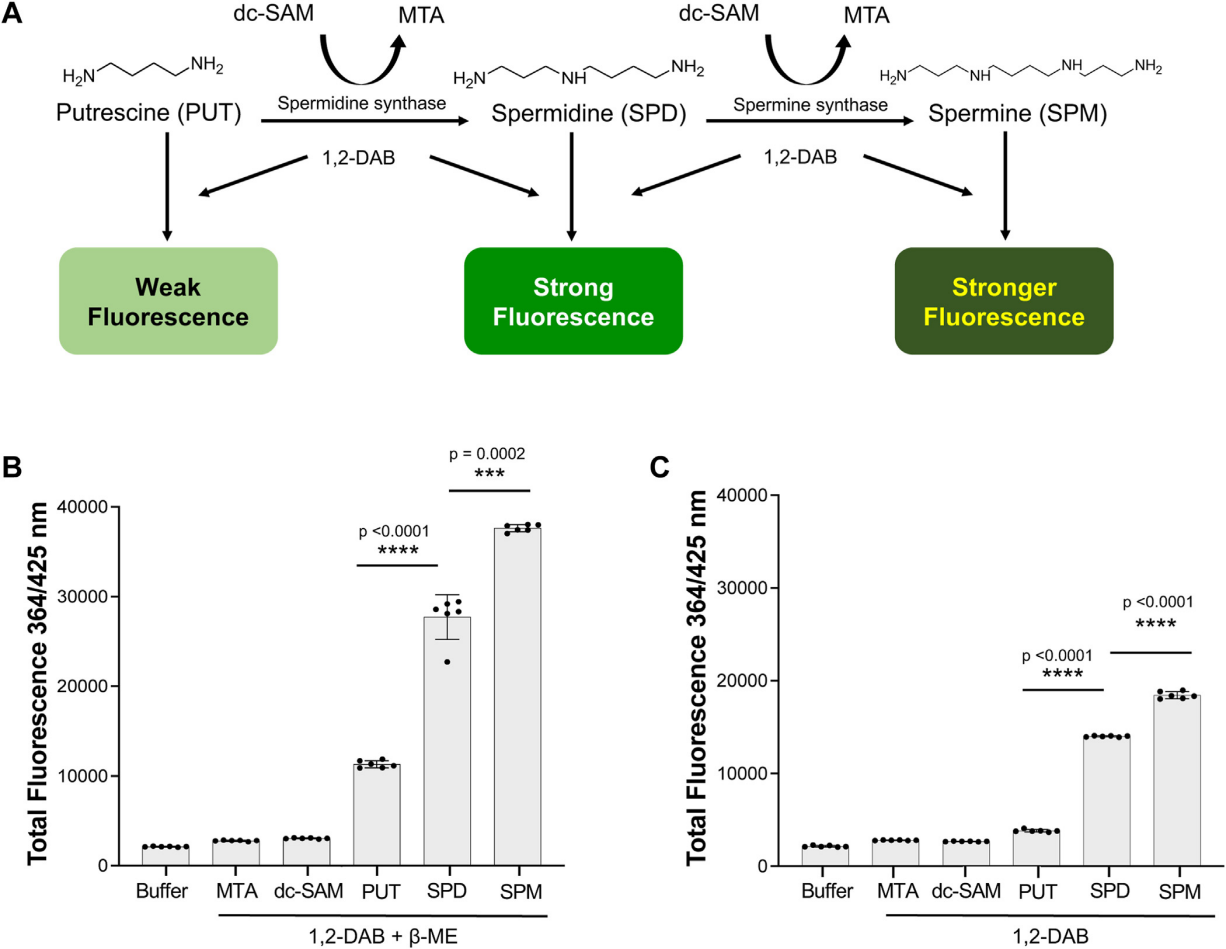
**Application of 1,2 DAB/β-ME based fluorescent assay to monitor aminopropyl transferase activity.***A*, schematic representation of the APT reaction and the detection of the products, spermidine or spermine, following the conversion of either putrescine or spermidine in the presence of dc-SAM, respectively, and subsequent interaction with 1,2 DAB to produce fluorescent adducts. Structures of the polyamines putrescine (PUT), spermidine (SPD), and spermine (SPM) are shown. *B* and *C*, total fluorescence intensities (λ_ex_ = 364 nm and λ_em_ = 425 nm) following incubation of 100 μM of either MTA, dc-SAM, putrescine, spermidine, or spermine with the APT reaction buffer containing 1,2-DAB in the absence (*B*), or presence of β-mercaptoethanol (*C*). The data are presented from three independent experiments performed in duplicates, and values are mean ± SD. β-ME, beta-mercaptoethanol; 1,2-DAB, 1,2-diacetyl benzene; dc-SAM, decarboxylated SAM; MTA, 5′-methylthioadenosine.

Previous studies by Medici *et al.* (22) and Choi *et al.* (23) have demonstrated the interaction of 1,2-DAB with the primary amines of molecules such as tyramine, gamma-aminobutyric acid, and ethanolamine, but not those primary amines attached to α-carboxylated compounds such as serine, leading to the formation of fluorescent adducts. These adducts could be detected using a plate reader with excitation and emission wavelengths of 364 nm and 425 nm, respectively. Therefore, we examined whether a DAB-mediated fluorescence interaction with the polyamines, putrescine, spermidine, and spermine could be used to measure the activity of APT enzymes, which catalyze the conversion of putrescine to spermidine (SPDS activity) or spermidine to spermine (SPMS activity). First, we compared the fluorescence intensity following incubation of DAB with these polyamines as well as cosubstrate, dc-SAM, or reaction product MTA in the absence or presence of beta-mercaptoethanol (β-ME) (Fig. 1, *A*–*C*). The reaction of spermidine with 1,2 DAB/β-ME yielded ∼4-fold higher fluorescence than that of putrescine with 1,2 DAB/β-ME (Fig. 1*B*). On the other hand, the reaction of spermine with 1,2 DAB/β-ME yielded ∼1.5-fold higher fluorescence than that of spermidine with 1,2 DAB/β-ME (Fig. 1*B*). The fluorescence intensities of dc-SAM, and MTA with either 1,2 DAB or 1,2 DAB/β-ME were similar to that observed with buffer control (Fig. 1, *B* and *C*). While the overall fluorescence signals were reduced when the polyamines reacted with a detection buffer lacking β-ME, the fold difference in the fluorescence signals between putrescine-spermidine d) and spermidine-spermine (∼1.5-fold) remained unchanged (Fig. 1*C*).

We next measured the total fluorescence (364/425 nm) at different concentrations (10–100 μM) of putrescine, spermidine, and spermine over time (Fig. S1, *A*–*C*). All the tested concentrations of putrescine, spermidine, and spermine showed a linear increase in the total fluorescence intensity upon reaction with 1,2-DAB/β-ME during the first 60 min, after which the fluorescence signal reached a plateau. At 60 min, the ratio of fluorescent intensity between spermidine and putrescine was ∼ 4 fold, whereas that between spermine and spermidine was ∼1.5 fold (Fig. S1*D*).

**Figure 2:**
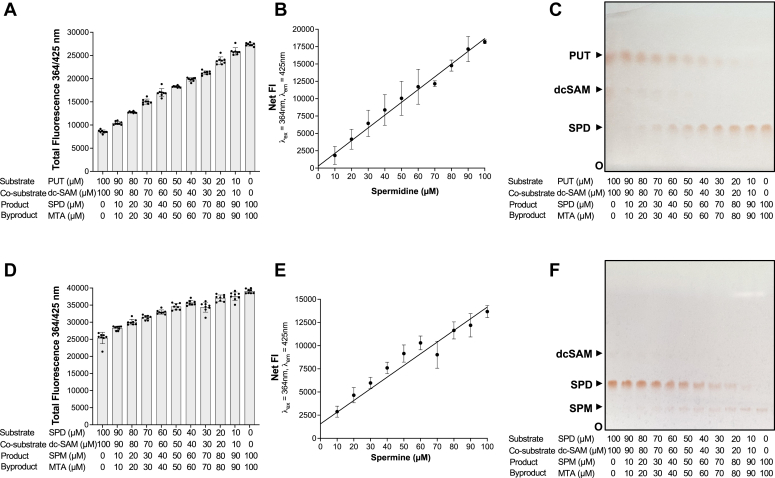
**Changes in the ratios of putrescine-spermidine and spermidine-spermine produce quantitative changes in 1,2-DAB/β-ME fluorescence.***A*, putrescine, dcSAM, spermidine, and MTA were mixed in different ratios as indicated in the figure (decreasing concentration of putrescine + dcSAM and increasing concentrations of spermidine + MTA), and the total PUT + dcSAM + SPD + MTA concentration was maintained at 200 μM. The total fluorescence intensity was measured at 364 nm (excitation)/425 nm (emission) after 1 h of incubation with 1,2-DAB/β-ME. *B*, the net fluorescent intensity (FI) was calculated from data in Fig. 2*A*. A linear increase in net fluorescence intensity of spermidine with increasing concentration was observed. *C*, TLC showing the separation of different fractions containing decreasing concentrations of putrescine + dcSAM and increasing concentrations of spermidine + MTA as shown in the figure. *D*, spermidine and spermine were mixed in different ratios as indicated in the figure (decreasing concentration of spermidine + dcSAM and increasing concentrations of spermine + MTA), and the total SPD + dcSAM + SPM + MTA concentration was maintained at 200 μM. The total fluorescence intensity was measured at 364 nm (excitation)/425 nm (emission) after 1 h of incubation with 1,2-DAB/β-ME. *E*, the net fluorescent intensity (FI) was calculated from data in Fig. 2*D*. A linear increase in net fluorescence intensity of spermine with increasing concentration was observed. *F*, TLC showing the separation of different fractions containing decreasing concentrations of spermidine + dcSAM and increasing concentrations of spermine + MTA as shown in the figure. The data are from three independent experiments conducted in triplicates, with error bars denoting mean ± standard error. 1,2-DAB, 1,2-diacetyl benzene; dc-SAM, decarboxylated SAM; β-ME, beta-mercaptoethanol; MTA, 5′-methylthioadenosine.

To mimic APT activities from fluorescence emission data and to estimate the conversion rates in enzyme reactions, standard curves were generated using variable ratios of the substrate and product (putrescine and spermidine or spermidine and spermine) (Fig. 2). To test whether the formation of spermidine in the Spe3-mediated reaction, and spermine in the Spe4-mediated reaction depicts a linear trend, two different standard curves were generated, one for each enzyme, by setting up mock reactions. The first set of mock reactions were conducted for Spe3 by decreasing the concentrations of putrescine (substrate) and dc-SAM (cosubstrate) and increasing the concentrations of spermidine (product) and MTA (by product) in the range of 100 to 0 μM, as shown in Fig. 2*A*. Net fluorescence intensities of increasing spermidine in the mixed putrescine/spermidine reactions were calculated after background correction with the fluorescence value of the 0 μM spermidine, μM MTA, 100 μM putrescine, and 100 μM dc-SAM from the data in Fig. 2*A*. A linear increase in fluorescence is observed with increasing concentrations of spermidine (Fig. 2*B*). To confirm whether the increase in fluorescence observed in Fig. 2, *A* and *B* is indeed due to an increase in spermidine concentration, the samples were analyzed by running on a TLC plate (500 μm) and stained with 0.2% ninhydrin (Fig. 2*C*). The second set of mock reactions were conducted for Spe4 by decreasing the concentrations of spermidine (substrate) and dc-SAM (cosubstrate) and increasing the concentrations of spermine (product) and MTA (by product) in the range of 100 to 0 μM (Fig. 2*D*). Net fluorescence intensities of increasing spermine in the mixed spermidine/spermine reactions were calculated after background correction with the fluorescence value of the 0 μM spermine, 0 μM MTA, 100 μM spermidine, and 100 μM dc-SAM from data in Fig. 2*D*. A linear increase in fluorescence is observed with increasing concentrations of spermine (Fig. 2*E*). The reactions performed in Fig 2*D* were also analyzed by running on a TLC plate (500 μm) and stained with 0.2% ninhydrin (Fig. 2*F*). These data demonstrate a direct correlation between fluorescence intensity and the rate of conversion of the substrate to the product (Fig. 2).

The ability to differentiate between putrescine, spermidine, and spermine using 1,2-DAB, makes the DAB-APT assay suitable for measuring APT enzyme activities of SPDSs and SPMSs.

### Structures of fluorescent putrescine-DAB, spermidine-DAB, and spermine-DAB adducts were determined to be substituted 1,3-dimethyl isoindoles using mass spectrometry (MS) analysis

Putrescine, spermidine, and spermine were incubated in the presence of 1,2-DAB in a buffer solution, and the resulting fluorescent complexes were analyzed using flow injection analysis MS (FIA-MS) and liquid chromatography high resolution mass spectrometry (LC-HRMS), both with tandem mass spectrometry (MS/MS) fragmentation. Primary [M + H]^+^ ions were identified from full scan data, and their respective MS/MS fragmentation patterns were obtained (Figs. S2–S7). For all three polyamines, the primary mass ion corresponded to a substituted 1,3-dimethyl isoindole. Similar fluorescent substituted 2H-isoindoles (24) and 1,3-dimethyl isoindoles (23) have been shown to be produced by the combination of phthalaldehyde or 1,2-DAB and various nucleophilic reagents, including tyramine, gamma-aminobutyric acid, ethanolamine, and mercaptoethanol. The structure of the observed polyamine-DAB adduct depended upon the structure of the polyamine used (Figs. S2–S7). Putrescine reacted with 1,2-DAB to yield a fluorescent cyclic putrescine-DAB adduct (HRMS calculated for C_14_H_19_N_2_ [M + H]^+^ = 215.1543, observed [M + H]^+^ = 215.1545). Spermidine and spermine also provided fluorescent cyclic adducts spermidine-DAB adduct (HRMS calculated for C_17_H_26_N_3_ [M + H]^+^ = 272.2121, observed [M + H]^+^ = 272.2124) and spermine-DAB adduct (HRMS calculated for C_20_H_33_N_4_ [M + H]^+^ = 329.2700, observed for [M + H]^+^ = 329.2701). However, oxidized versions, spermidine-DAB adduct N-oxide (HRMS calculated for C_17_H_26_N_3_O_1_ [M + H]^+^ = 288.2070, observed for [M + H]^+^ = 288.2072) and spermine-DAB adduct N-oxide (HRMS calculated for C_20_H_33_N_4_O_1_ [M + H]^+^ = 345.2649, observed for [M + H]^+^ = 345.2651), were also observed. The oxidation was determined to have occurred on the primary amine *via* analysis of the MS/MS fragmentation pattern. It is worth noting that we did not observe the oxidized version of the putrescine-DAB adduct, which does not possess a primary amine.

**Figure 3:**
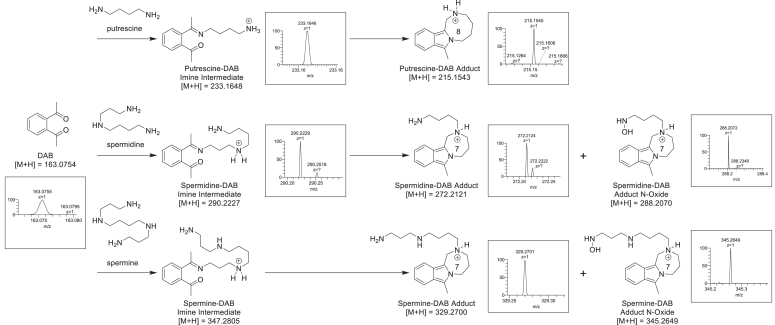
**Putrescine, spermidine, and spermine form fluorescent adducts with 1,2-DAB.** Proposed mechanism of formation of fluorescent 1,3-dimethyl isoindole adducts. Formation of 1,2-DAB-polyamine adducts from 1,2-DAB and putrescine, spermidine, and spermine. 1,2-DAB, 1,2-diacetyl benzene.

The formation of the polyamine-DAB adducts can be rationalized using a simple, well-precedented (24) arrow-pushing mechanism (Fig. 3). Adduct formation is initiated by the reaction of a primary amine and a ketone to provide a Schiff base intermediate A. Here, the polyamine is putrescine, but the mechanism is the same for spermidine and spermine, with the assumption that only a primary amine can form the Schiff base. Remarkably, these Schiff base intermediates were also observed in the LC-HRMS data: putrescine-DAB intermediate (HRMS calculated for C_14_H_21_N_2_O_1_ [M + H]^+^ = 233.1648, observed for [M + H]^+^ = 233.1646) and spermidine-DAB intermediate (HRMS calculated for C_17_H_28_N_3_O_1_ [M + H]^+^ = 290.2227, observed for [M + H]^+^ = 290.2227) (Figs. S2–S3). Cyclization *via* intramolecular reaction between the nucleophilic enamine nitrogen and remaining ketone gives amino alcohol intermediate B, which eliminates to form intermediate C. An intramolecular Michael-type cyclization between the remaining putrescine amine and the electrophilic methylene provides the putrescine-DAB adduct. The spermidine-DAB and spermine-DAB adducts are formed as a result of cyclization with a secondary amine.

### Activity of *S. cerevisiae* APT enzymes, Spe3, and Spe4 using the DAB-APT fluorescence assay

**Figure 4:**
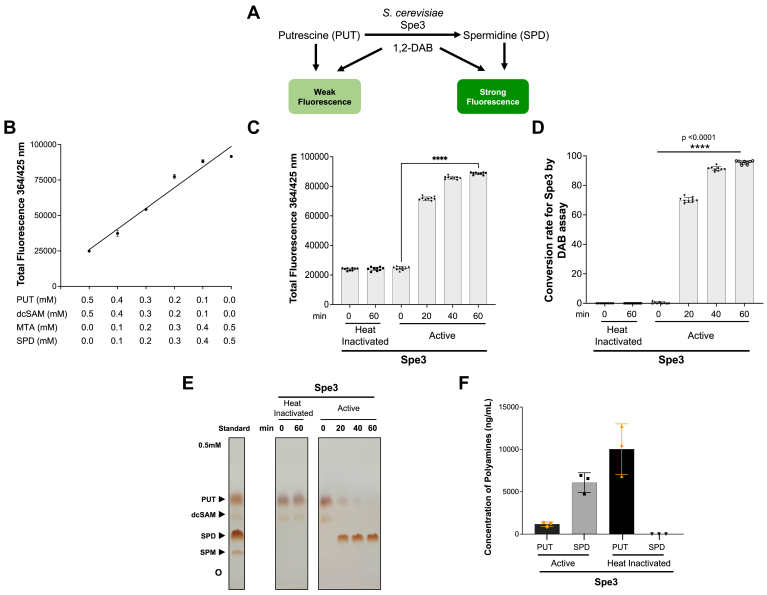
**Application of the DAB-APT fluorescence assay to determine the activity *Saccharomyces cerevisiae* spermidine synthase Spe3.***A*, schematic representation of the enzymatic reaction catalyzed by Spe3 along with and the anticipated fluorescence signals using the DAB-APT assay. *B*, standard curve was generated by mixing different ratios of putrescine, dcSAM, spermidine, and MTA (decreasing concentration of putrescine + dcSAM and increasing concentrations of spermidine + MTA) as indicated in the figure, and the total PUT + dcSAM + SPD + MTA concentration was maintained at 1 mM. The total fluorescence intensity was measured at 364 nm (excitation)/425 nm (emission) after 1 h of incubation with 1,2-DAB/β-ME. A linear increase in fluorescence is observed with saturation around 0.1 mM of PUT, dcSAM, and 0.4 mM of SPD + MTA. *C*, spermidine synthase assays were conducted using affinity-purified recombinant MBP-Spe3 (20 ng/μl), heat-inactivated MBP-Spe3 and 0.5 mM of putrescine, and dc-SAM as substrate and co-substrate, respectively, at 37 °C for 0 to 60 min. The total fluorescence intensities of the reactions catalyzed by heat-inactivated or active MBP-Spe3 at 0 min and 60 min were measured at 364 nm (excitation)/425 nm (emission) after 1 h of incubation with 1,2-DAB/β-ME buffer. *D*, conversion rates of the substrates putrescine and dcSAM by heat-inactivated and active MBP-Spe3 as determined by DAB assay is shown as percentage of spermidine formed, from data in Fig. 4*C*. Data presented as mean ± SD from three independent experiments, each conducted in triplicate. *E*, TLC showing the reactions performed in Fig. 4*C*, confirming the formation of the product spermidine by active MBP-Spe3. *F*, concentration of polyamines (substrate putrescine and the product spermidine) in reactions catalyzed by active and heat-denatured MBP-Spe3 after 60 min of reactions as determined using LC-MS. 1,2-DAB, 1,2-diacetyl benzene; APT, aminopropyl transferase; β-ME, beta-mercaptoethanol; MTA, 5′-methylthioadenosine; dc-SAM, decarboxylated SAM; MBP, maltose-binding protein.

**Figure 5:**
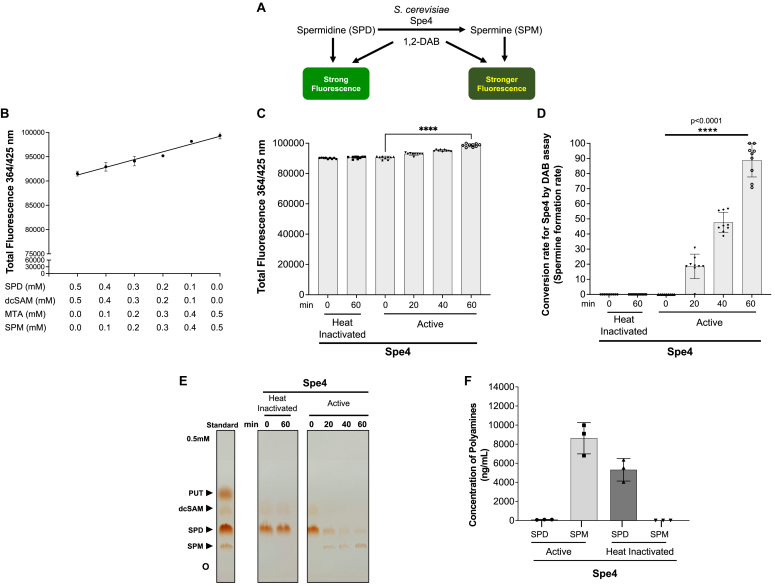
**Application of the DAB-APT fluorescence assay to determine the activity of *Saccharomyces cerevisiae* spermine synthase Spe4.***A*, schematic representation of the enzymatic reaction catalyzed by Spe4, along with the anticipated fluorescence signals using the DAB-APT assay. *B*, standard curve was generated by mixing different ratios of spermidine, dcSAM, spermine, and MTA (decreasing concentration of spermidine + dcSAM and increasing concentrations of spermine + MTA) as indicated in the figure, and the total SPD + dcSAM + SPM + MTA concentration was maintained at 1 mM. The total fluorescence intensity was measured at 364 nm (excitation)/425 nm (emission) after 1 h of incubation with 1,2-DAB/β-ME. A linear increase in fluorescence is observed. *C*, spermine synthase assays were conducted using recombinant MBP-Spe4 (20 ng/μl), heat-inactivated MBP-Spe4, and 0.5 mM of spermidine and dc-SAM as substrate and cosubstrate, respectively, at 37 °C for 0 to 60 min. The total fluorescence intensities of the reactions catalyzed by heat-inactivated or active MBP-Spe4 at 0 min and 60 min were measured at 364 nm (excitation)/425 nm (emission) after 1 h of incubation with 1,2-DAB/β-ME buffer. *D*, conversion rates of the substrates spermidine, and dcSAM by heat inactivated and active MBP-Spe4 is shown as percentage of spermine formed, from data Fig. 5*C*. Data presented as mean ± SD from three independent experiments, each conducted in triplicate. *E*, TLC showing the reactions performed in Fig. 5*C*, confirming the formation of the product spermine by active Spe4. *F*, concentration of polyamines (substrate spermidine and the product spermine) in reactions catalyzed by active and heat denatured Spe4 after 60 min of reaction determined using LC-MS. 1,2-DAB, 1,2-diacetyl benzene; APT, aminopropyl transferase; β-ME, beta-mercaptoethanol; MTA, 5′-methylthioadenosine; dc-SAM, decarboxylated SAM; MBP, maltose-binding protein.

To validate the use of the DAB-APT assay for measurement of APT enzyme activity, we first examined the activity of the yeast Spe3 (Fig. 4*A*) and Spe4 (Fig. 5*A*), which have been well characterized at the genetic level and shown to catalyze either putrescine to spermidine or spermidine to spermine, respectively (18, 25). Standard curves were generated using reactions containing either decreasing concentrations of putrescine, dc-SAM (range: 0.5 mM to 0 mM), and increasing concentrations of spermidine, MTA (range: 0 mM–0.5 mM) (Fig. 4*B*), or decreasing concentrations of spermidine, dc-SAM (range: 0.5 mM–0 mM), and increasing concentrations of spermine, MTA (range: 0 mM–0.5 mM) (Fig. 5*B*). These standard curves were used for determination of the conversion rates (spermidine or spermine formation rates) of Spe3 and Spe4 enzymes. Spe3 and Spe4 were expressed in *Escherichia coli* as N-terminal fusion proteins with the maltose-binding protein (MBP) (Fig. S8, *A*–*B*), affinity-purified, and used in enzyme reactions in the presence of either putrescine (0.5 mM) or spermidine (0.5 mM) and the cosubstrate dc-SAM (0.5 mM). The reactions were performed with either active or heat inactivated enzymes (Spe3 and Spe4), and the reactions were stopped by the addition of the detection buffer consisting of 1,2 DAB/β-ME in sodium tetraborate buffer (pH 9.6) and incubated at 22 °C (room temperature) for 60 min. Fluorescence was measured using a plate reader with excitation and emission at wavelengths of 364 nm and 425 nm, respectively. The active yeast Spe3 catalyzed the conversion of putrescine and dc-SAM to form spermidine over time (Fig. 4, *C*–*E*), with complete conversion to spermidine achieved in ∼40 min, whereas the heat inactivated Spe3 failed to convert putrescine into spermidine (Fig. 4*C*). The active yeast Spe4 catalyzed the conversion of spermidine and dc-SAM to form spermine (Fig. 5, *C*–*E*), with complete conversion to spermine achieved in ∼60 min. To validate the products of the reactions catalyzed by Spe3 and Spe4, reaction mixtures were separated byTLC (Figure 4, Figure 5*E* and 5*E*), and the levels of putrescine in Fig. 4*E* and spermidine in Fig. 5*E* were quantified using ImageJ (https://imagej.net/ij/) and used for the calculation of putrescine (Spe3-mediated conversion rate) and spermidine consumption rates (Spe4-mediated conversion rate) (Fig. S10, *A*–*B*). The levels of spermidine determined using the DAB-APT assay were similar to those determined using quantification of the spermidine spot on TLC plate (Figs. 4, *D*–*E*; S10*A*), whereas the levels of spermine determined from DAB-APT assay were slightly lower than those determined using the spermine spot on TLC plate (Fig. 5, *D*–*E*, S10*B*). Further validation of the products of the reactions was achieved using LC-MS analysis (Figure 4, Figure 5*F* and 5*F*). Together, these data demonstrate that the DAB-APT assay is a reliable assay for measuring the activity of spermidine and spermine synthases.

### Dual activity of *P. falciparum* spermidine synthase using the DAB-APT fluorescence assay

**Figure 6:**
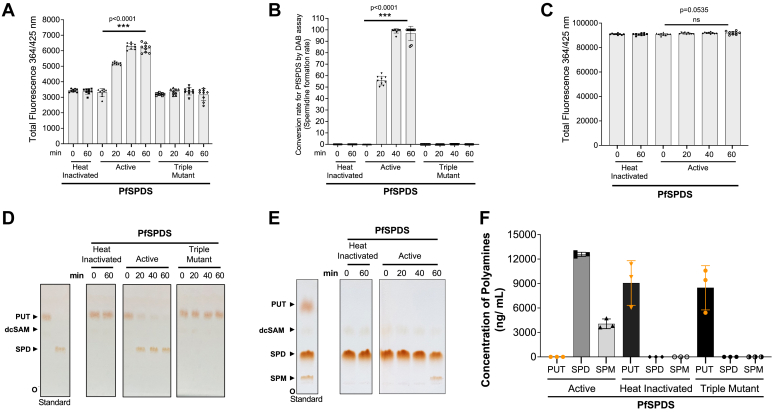
**Application of the DAB-APT fluorescence assay to determine the activity of the *Plasmodium falciparum* PfSPDS enzyme.***A*, spermidine synthase assays were conducted using affinity-purified recombinant MBP-PfSPDS (20 ng/μl), heat-inactivated MBP-PfSPDS, MBP-triple mutant PfSPDS (MBP-PfSPDS^D127A,E147A,D196A^), and 0.5 mM of putrescine and dc-SAM as substrate and cosubstrate, respectively, at 37 °C for 0 to 60 min. The total fluorescence intensities of the above-mentioned reactions at 0 min and 60 min were measured at 364 nm (excitation)/425 nm (emission) after 1 h of incubation with 1,2-DAB/β-ME buffer. *B*, conversion rates of the substrates putrescine, and dcSAM by heat-inactivated, active and triple mutant MBP-PfSPDS (is shown as percentage of spermidine formed, from data in Fig. 6*A*. Data presented as mean ± SD from three independent experiments, each conducted in triplicate. *C*, spermine synthase assays were conducted using heat-inactivated or active MBP-PfSPDS, with 0.5 mM of spermidine and 0.75 mM of dc-SAM as substrate and cosubstrate, respectively, at 37 °C for 0 to 60 min. The total fluorescence intensities of the reactions catalyzed by heat-inactivated or active MBP-PfSPDS at 0 min and 60 min were measured at 364 nm (excitation)/425 nm (emission) after 1 h of incubation with 1,2-DAB/β-ME buffer. Data presented as mean ± SD from three independent experiments, each conducted in triplicate. *D*, TLC showing the reactions performed in Fig. 6*A*, confirming the formation of the product spermidine by active MBP-PfSPDS. *E*, TLC showing the reactions performed in Fig. 6*C*, showing the formation of the product spermine by active MBP-PfSPDS at 60 min. *F*, concentration of polyamines (substrate putrescine and the products spermidine, and spermine) in a reaction catalyzed by active, heat-denatured, and the triple mutant PfSPDS after 90 min, as determined by LC-MS. 1,2-DAB, 1,2-diacetyl benzene; APT, aminopropyl transferase; β-ME, beta-mercaptoethanol; MTA, 5′-methylthioadenosine; dc-SAM, decarboxylated SAM; MBP, maltose-binding protein; PfSPDS, *P. falciparum* spermidine synthase.

We further examined the use of the DAB-APT assay to measure the activity of the *P. falciparum* spermidine synthase (PfSPDS). This enzyme has been shown to have a primary function as a SPDS but can also convert spermidine to spermine, with the SPMS activity accounting for only a small but significant amount of spermine found in *P. falciparum*-infected erythrocytes (26, 27, 28, 29). The catalytic site residues are conserved between PfSPDS and APT enzymes from other organisms like *E. coli*, *S. cerevisiae*, and *Homo sapiens* (Fig. S9). PfSPDS, as well as the triple mutant PfSPDS^D127A,E147A,D196A^, with the three key catalytic residues, D127, E147, and D196 mutated to alanine, were expressed in *E. coli* as N-terminal fusion proteins with MBP (Fig. S8, *C,* and *F*), affinity purified, and used in enzyme reactions in the presence of putrescine (0.5 mM) and the cosubstrate dc-SAM (0.5 mM). The reactions were performed with active, heat inactivated, and triple mutant PfSPDS recombinant proteins, and the reactions were stopped by the addition of the detection buffer consisting of 1,2 DAB/β-ME in sodium tetraborate buffer (pH 9.6) and incubated at 22 °C (room temperature) for 60 min. Fluorescence was measured using a plate reader with excitation and emission spectra at wavelengths of 364 nm and 425 nm, respectively. The active PfSPDS catalyzed the conversion of putrescine and dc-SAM to form spermidine (Fig. 6, *A*, *B* and *D*) over time, with complete conversion to spermidine achieved in ∼40 min, whereas the heat inactivated, and triple mutant PfSPDS failed to convert putrescine into spermidine (Fig. 6, *A*, *B* and *D*). These reaction mixtures were further separated by TLC to visualize and quantify the substrate and product of the reactions (Fig. 6*D*) and estimate the rate of catalysis (PfSPDS-mediated conversion rate) (Fig. S10*C*). Our data showed that the levels of spermidine determined using the DAB-APT assay were similar to those determined by TLC. The SPMS activity of PfSPDS was also tested by conducting enzyme reactions with PfSPDS as well as a heat-inactivated enzyme (negative control) in the presence of spermidine and the cosubstrate dc-SAM. Unlike with the yeast Spe4 enzyme, the DAB-APT assay using active PfSPDS showed little to no significant increase in fluorescence over that of the spermidine substrate, suggesting that the SPMS activity of PfSPDS is either weak or absent. No conversion of spermidine to spermine in reactions containing the heat inactivated PfSPDS enzyme using the DAB-APT assay (Fig. 6, *C* and *E*). Interestingly, when the same enzyme reaction mixtures were analyzed by TLC (Fig. 6*E*) and MS (Fig. 6*F*), a small amount of spermine was detected after a 60 min enzyme incubation, with an estimated ∼20% of the spermidine substrate converted into spermine (Fig. 6, *E* and *F*, S10*D*). These data suggest that the DAB-APT assay is suitable for measuring the formation of spermine from spermidine only for APT enzymes with strong SPMS activity.

### Kinetics of Spe3, Spe4, and PfSPDS using the DAB-APT fluorescence assay

**Figure 7:**
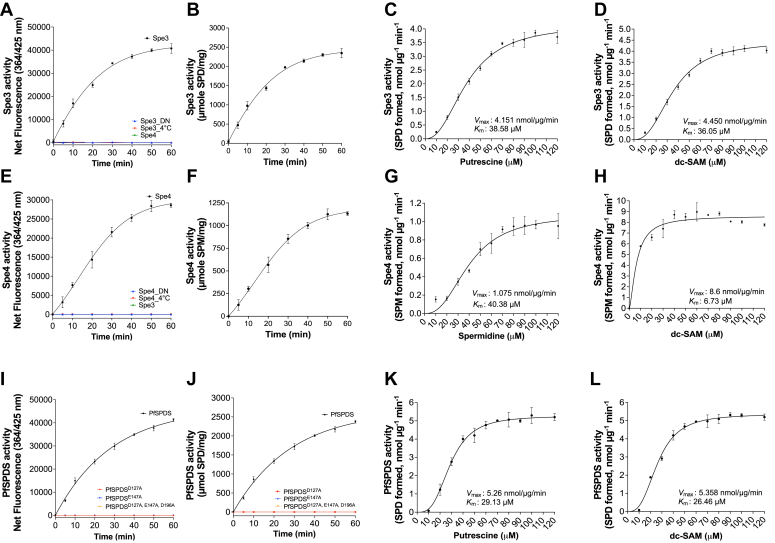
**Application of the DAB-APT fluorescence assay to determine the activities and kinetics of *Saccharomyces cerevisiae* spermidine synthase Spe3, spermine synthase Spe4, and *Plasmodium falciparum* spermidine synthase.***A*, spermidine synthase assays were conducted using affinity-purified recombinant MBP-Spe3 (20 ng/μl), heat-denatured Spe3 (Spe3_DN), and 100 μM of putrescine and dc-SAM as substrate and cosubstrate, respectively, at 37 °C for 0 to 60 min. A parallel reaction at 4 ˚C (Spe3 4 °C) served as a control, the same reaction was also performed at 4 °C. Purified MBP-Spe4, which lacks the ability to convert putrescine to spermidine, was included as an additional control. The spermidine synthase activity of MBP-Spe3, MBP-Spe4, and respective controls is depicted as net fluorescence intensity over time. *B*, spermidine synthase APT activity of yeast SPE3 calculated from Fig. 7*A* and shown as a function of time. *C* and *D*, kinetics of the Spe3 spermidine synthase activity as a function of putrescine (*C*) and dc-SAM (*D*) concentrations. Spe3-APT assays were performed with 20 ng/μl of MBP-Spe3 and varying concentrations of either putrescine or dc-SAM at 37 °C for 60 min. *E*, spermine synthase assays mediated by Spe4 were performed using affinity-purified recombinant MBP-Spe4 (20 ng/μl), heat-denatured SPE4 (Spe4_DN), along with 100 μM of spermidine and dc-SAM as substrates at 37 °C for 0 to 60 min. A control reaction was conducted at 4 °C (Spe 4 °C). Spe3, which lack the ability to convert spermidine to spermine, was used as an additional control. The spermine synthase activity of MBP-Spe4, MBP-Spe3, and controls is depicted as net fluorescence intensity over time. *F*, spermine synthase activity of yeast Spe4 calculated from data in Fig. 7*E* and represented as molar amounts of spermidine formed over time. *G* and *H*, kinetics of the Spe4 spermine synthase activity as a function of spermidine (*G*) and dc-SAM (*H*) concentrations. Spe4-APT assays were performed with 20 ng/μl of MBP-Spe3 and varying concentrations of either spermidine or dc-SAM at 37 °C for 60 min. *I*, spermidine synthase reactions were conducted using affinity-purified recombinant MBP-PfSPDS (20 ng/μl), mutant enzymes (MBP-PfSPDS^D127A^, MBP-PfSPDS^E147A^, MBP-PfSPDS^D127A,E147A,D196A^), and 100 μM of putrescine and dc-SAM as substrate and cosubstrate, respectively, at 37 °C for 0 to 60 min. The spermidine synthase activity of MBP-PfSPDS, PfSPDS mutants, and controls is depicted as net fluorescence intensity over time. *J*, spermidine synthase activity of PfSPDS and mutants was calculated from data in Fig. 7*I* and shown as a function of time. *K* and *L*, kinetics of PfSPDS spermidine synthase activity as a function of putrescine (*K*) or dc-SAM (*L*) concentrations. PfSPDS-APT assays were conducted with PfSPDS and varying concentrations of either putrescine or dc-SAM at 37 °C for 60 min. *V*_*max*_ and *K*_*m*_ values were determined using Michaelis–Menten kinetics in GraphPad Prism. Data are presented as mean ± SD from three independent experiments, each conducted in triplicate. 1,2-DAB, 1,2-diacetyl benzene; APT, aminopropyl transferase; β-ME, beta-mercaptoethanol; MTA, 5′-methylthioadenosine; dc-SAM, decarboxylated SAM; MBP, maltose-binding protein

The DAB-APT fluorescence assay for detection of APT activity provides an attractive platform for screening of novel compound libraries in a high-throughput format. Since high-throughput screens are performed using substrate concentrations near the *K*_m_ values of the substrates, we determined the kinetic constants for the yeast Spe3, Spe4, and PfSPDS. Firstly, we tested whether the DAB-APT assay can be used with lower substrate concentrations, that is, 100 μM instead of 500 μM. A time-dependent increase in fluorescence was detected when Spe3, but not Spe4, was incubated with putrescine and dc-SAM, consistent with an increase in spermidine formation (Fig. 7*A*). As a control, no fluorescence signals could be detected using heat-denatured Spe3 (Spe3_DN) or in Spe3 reactions conducted at 4 °C (Fig. 7*A*). The molar amounts of spermidine formed in the Spe3-catalyzed reaction were calculated (Fig. 7*B*) from the net fluorescence data in Fig. 7*A*, using the standard curves shown in Fig. 2*B*. For Spe3, the *K*_m_ (38.6 μM) and *V*_max_ (4.2 nmol/μg/min) (n = 3) for putrescine as well as the *K*_m_ (36.1 μM) and *V*_max_ (4.5 nmol/μg/min) (n = 3) for dc-SAM were determined (Fig. 7, *C* and *D*) using Michaelis–Menten kinetics. Similarly, when yeast Spe4 was used in the APT reaction with spermidine and dc-SAM as substrate and cosubstrate (Fig. 7*E*), respectively, a time-dependent increase in fluorescence resulting from the formation of spermine was detected using active but not heat-inactivated enzyme (Fig. 7*E*). The molar amounts of spermine formed in the Spe4 reaction (Fig. 7*F*) were calculated from the net fluorescence data in Fig. 7*E*, using the standard curves shown in Fig. 2*E*. Characterization of the kinetic parameters of Spe4 using the DAB-APT assay yielded a *K*_m_ of 40.4 μM and a *V*_max_ of 1.1 nmol/μg/min for spermidine and a *K*_m_ of 6.7 μM and a *V*_max_ of 8.6 nmol/μg/min for dc-SAM (Fig. 7, *G* and *H*). In the case of PfSPDS, a time-dependent increase in fluorescence was only observed with active PfSPDS, but not with the mutant enzymes (PfSPDS^D127A^, PfSPDS^E147A^, and PfSPDS^D127A,E147A,D196A^) (Fig. 7*I*). The molar amounts of spermidine formed in the PfSPDS-catalyzed reaction were calculated (Fig. 7*J*) from the net fluorescence data in Fig. 7*I*. For PfSPDS, the *K*_m_ (29.13 μM) and *V*_max_ (5.26 nmol/μg/min) (n = 3) for putrescine as well as the *K*_m_ (26.46 μM) and *V*_max_ (5.358 nmol/μg/min) (n = 3) for dc-SAM were determined (Fig. 7, *K* and *L*) using Michaelis–Menten kinetics.

### The DAB-APT assay is amenable to high throughput screening

**Figure 8:**
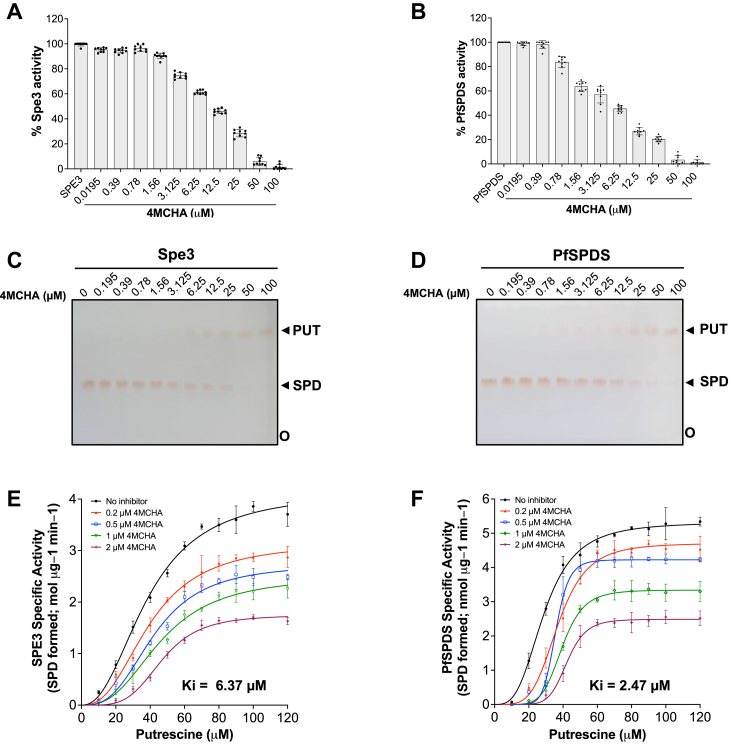
**Inhibition of *Saccharomyces cerevisiae* Spe3 and *Plasmodium falciparum* PfSPDS activity by 4MCHA.***A*, A dose-dependent decrease in the activity of MBP-Spe3 with increasing concentrations of 4MCHA (0.0195μM–100 μM). *B*, a dose-dependent decrease in the activity of MBP-PfSPDS with increasing concentrations of 4MCHA (0.0195μM–100 μM). The data are from three independent experiments conducted in triplicates, with error bars denoting mean ± standard error. *C*, TLC of the reactions performed in Fig. 8*A*, showing a dose-dependent decrease in the activity of Spe3. *D*, TLC of the reactions performed in Fig. 8*B*, showing a dose-dependent decrease in the activity of PfSPDS. *E* and *F*, determination of inhibition constants (Ki) values for inhibition of *S. cerevisiae* Spe3 (*B*) and *P. falciparum* PfSPDS (*C*) spermidine synthase activity by 4MCHA. All data are presented as mean ± SD from three independent experiments, each conducted in triplicate. MBP, maltose-binding protein; 4MCHA, 4-methylcyclohexylamine; PfSPDS, *P. falciparum* spermidine synthase.

The adaptability of the 1,2 DAB/β-ME-APT assay to a 96-well plate format for conducting inhibitor screening in high throughput screening platforms was evaluated using trans-4-methylcyclohexylamine (4MCHA), a known potent inhibitor of SPDS. 4MCHA has been demonstrated to compete with putrescine binding sites on SPDS enzymes (30). The putrescine to spermidine APT activity of *S. cerevisiae* Spe3 and PfSPDS was determined in 96-well format in a 100 μl volume in the absence or presence of increasing concentrations of 4MCHA (0–100 μM), and spermidine formation was determined by measuring fluorescence intensity using the DAB-APT assay (Fig. 8, *A* and *B*). Samples from the same enzyme inhibition reaction mixtures analyzed by the DAB-APT assay (Fig. 8, *A* and *B*) were further separated by TLC to visualize the substrate and product of the reactions (Fig. 8, *C* and *D*). The inhibition constants for the compound were determined to be *K*_*i*_ = 6.4 μM for Spe 3 and *K*_*i*_ = 2.5 μM for PfSPDS enzymes (Fig. 8, *E* and *F*). The kinetic parameters *K*_m_ and *V*_max_ of the inhibition curves obtained with different concentrations of 4MCHA indicate a competitive inhibition by 4MCHA, which supports the previous findings where 4MCHA has been demonstrated to compete with putrescine for binding to the enzyme (30). The amenability of the DAB-APT assay to high throughput screening was determined for the yeast Spe3 enzyme by calculating the signal to background (S/B) and *Z*′ score as detailed in the Methods. From these analyses, the S/B (∼4) and *Z*′ score (0.83) suggest that the assay is suitable for high-throughput screening and may be used to screen chemical libraries to search for novel compounds for various indications.

## Discussion

In this study, we describe a novel fluorescence assay utilizing 1,2-DAB and β-ME for the measurement of APT activity. This assay builds upon previous successful applications of DAB, notably in measuring the activity of phosphatidylserine decarboxylase enzymes, where DAB interacts specifically with ethanolamine but not serine (23). Our studies demonstrated that DAB interacts with the primary amines of polyamines—putrescine, spermidine, and spermine—to form fluorescent adducts, likely through a chemical process similar to that observed with ethanolamine (23). Although all three polyamines carry two primary amine groups available for reaction with 1,2-DAB, our data revealed differential fluorescence signals for putrescine, spermidine, and spermine (Fig. 1*B*). We hypothesize that the higher fluorescence signals observed with spermine (tetramine) *versus* spermidine (triamine) and putrescine (diamine) are due to the increased chain length of the polyamines, which separates the two primary amine groups and reduces potential steric hindrance, thereby enhancing fluorescence.

The addition of β-ME further enhanced the assay's sensitivity, enabling the detection of subtle changes in APT enzyme activity. This suggests a potential role for β-ME in facilitating the interaction between DAB and polyamines, thereby amplifying the fluorescence signal. Our findings underscore the importance of optimizing assay conditions to maximize both sensitivity and specificity, which are essential for accurate enzyme activity measurements.

To validate the DAB-based assay for APT enzyme activity, we employed an optimized TLC assay and MS alongside the fluorescence assay. The DAB-APT assay confirmed that in *S. cerevisiae*, Spe3 catalyzes the conversion of putrescine to spermidine but lacks SPMS activity, while Spe4 catalyzes the conversion of spermidine to spermine but lacks SPDS activity. However, the assay did not provide evidence of dual activity for the *P. falciparum* PfSPDS enzyme. While PfSPDS was clearly shown to convert putrescine to spermidine, in the presence of spermidine as a substrate, the DAB-APT assay failed to detect a significant change in fluorescence above that of spermidine alone (Fig. 6*C*). However, TLC and MS analyses demonstrated that PfSPDS does indeed convert spermidine to spermine, but this activity is very weak, with only ∼ 20% of the substrate being converted into the product after a 90 min reaction (Fig. 6, *E* and *F*). These findings suggest that while the DAB-APT assay can reliably be used to examine the activity of both spermidine and spermine synthases, the fact that the fluorescence of spermine is only 1.5-fold higher than that of spermidine indicates that only highly active enzymes, capable of converting more than 25% of spermidine to spermine, would be suitable for detection using this assay. On the other hand, because the fluorescence difference between putrescine and spermidine is more than 3-fold, the assay can reliably detect all SPDSs, even those with weak activity.

Our data using FIA-MS and LC-HRMS, both with MS/MS fragmentation, suggest that the structure of fluorescent adducts formed between putrescine, spermidine, spermine, and 1,2-DAB are likely substituted 1,3-dimethyl isoindoles. Similar structures have been reported in the literature (23). Interestingly, the resulting adduct structures varied depending on the polyamine involved. A fluorescent cyclic DAB-polyamine adduct was observed for putrescine ([M + H]^+^ = 215.1545), spermidine ([M + H]^+^ = 272.2124), and spermine ([M + H]^+^ = 329.2701). For spermidine and spermine, oxidized cyclic adducts ([M + H]^+^ = 288.2072 and 345.2651, respectively) were also observed. The formation of these adducts can be explained through a well-precedented mechanistic pathway involving Schiff base formation, cyclization, and an intramolecular Michael-type reaction.

The stability of the DAB-polyamine adducts follows a continuum (DAB-putrescine < DAB-spermidine < DAB-spermine), which correlates with the observed fluorescence intensities. The MS/MS fragmentation patterns confirmed that the more stable cyclic structures are preferentially observed, aligning with the fluorescence data. Every step in the polyamine-DAB adduct-forming mechanism is reversible. The putrescine-DAB adduct can occur as one cyclic isomer, an 8-membered ring. The spermine-DAB adduct can occur as a 7-, 12-, or 16-membered ring, depending on which amine participates in the final cyclization step. However, only the 7-membered ring is observed *via* MS/MS, which is an indication of its relative stability. Because unsymmetrical spermidine has two different primary amine ends, the spermidine-DAB Adduct can occur as a 7-, 8-, or 12-membered ring. As for spermine, only the 7-membered ring is observed *via* MS/MS. This was surprising, since the 8-membered ring of the putrescine-DAB adduct is observed *via* MS/MS. Because of the relative stability and the reversibility of formation of the polyamine-DAB adducts, the 7-membered spermine-DAB adduct is more fluorescent than the 8-membered putrescine-DAB adduct. Similarly, because either primary amine end of nonsymmetrical spermidine is equally likely to form the initial Schiff base, it is possible to rationalize the observed intermediate level of fluorescence intensity of the spermidine-DAB adduct as resulting from, relative to spermine, less of the stable 7-membered ring being formed.

Attempts to purify the fluorescent adduct from the reaction of putrescine with DAB or to synthesize it *via* a more controlled sequence of reactions were not successful. Initial experiments using NMR to verify the polyamine-DAB adduct structures have been inconclusive. We conclude that the likely cyclic isoindole products are stable enough for robust fluorescence assays and for analysis by MS, but they are minor components of the total assay reaction products and are unstable toward isolation. Further investigations using a combination of advanced analytical techniques and potentially novel synthetic approaches may help achieve a more accurate characterization of the adducts.

Importantly, our assay exhibited suitability for inhibitor screening in high-throughput platforms. By utilizing known inhibitors, such as 4MCHA, we successfully determined their inhibitory potency against APT enzymes (Fig. 8). The competitive inhibition observed with 4MCHA underscores the potential of our assay in identifying novel inhibitors targeting polyamine metabolism, with implications for therapeutic intervention in diseases such as cancer and parasitic infections.

In conclusion, our study presents a robust and versatile fluorescence assay for accurately measuring APT enzyme activity. Despite the challenges in synthesizing the adducts, the assay's sensitivity, specificity, and suitability for high-throughput screening make it a valuable tool for drug discovery and polyamine metabolism research. We anticipate that our findings will pave the way for future studies exploring the role of APT enzymes in health and disease, as well as the development of novel therapeutics targeting polyamine metabolism pathways.

## Experimental procedures

### Materials

*S. cerevisiae SPE3* and *SPE4* and *P. falciparum* SPDS were codon-optimized for expression in *E. coli*, and cloned into pMAL-c4x-1-H(RBS) plasmid (GenScript). Putrescine (P5780-5G), spermidine (S0266-1G), spermine (S4264-1G), 5-deoxy-5-methylthioadenosine (260585), 1,2-DAB (242039) were purchased from Millipore Sigma. Dc-SAM was purchased from BOC Sciences, and 2-mercaptoethanol (1610710) was purchased from Bio-Rad.

### Expression and purification of MBP-tagged Spe3, Spe4, and PfSPDS

The *SPE3*-pMAL-c4x-1-H(RBS), *SPE4*-pMAL-c4x-1-H(RBS), and *PfSPDS*-pMAL-c4x-1-H(RBS) plasmids were transformed into *Rosetta (DE3) E. coli* cells (Thermo Fisher Scientific, 713973). Two clones from each transformation were selected and tested for the expression of MBP-tagged Spe3, Spe4, and PfSPDS. Briefly, each clone was inoculated into 5 ml of Luria broth (26) containing ampicillin (50 μg/ml) and incubated overnight at 37 °C in a shaker incubator (200 rpm). The following day, secondary cultures were inoculated with the overnight cultures and grown until *A*_*600*_ reached 0.6. Following this step, 0.5 mM IPTG was added and the cultures were shifted to 16 °C for 16 h. Cells were harvested by centrifugation at 5000*g* for 5 min, resuspended in 1X Laemmli sample buffer (1610737EDU; Bio-Rad), boiled at 95 °C for 10 min, and centrifuged again at 10,000 g for 5 min. Protein expression in uninduced and induced samples was checked by running the supernatants on 4-20% SDS-PAGE (4561096; Bio-Rad) followed Coomassie staining.

For the purification of recombinant MBP-tagged Spe3, MBP-Spe4, MBP-PfSPDS, 500 ml culture for each protein was grown in LB medium–containing ampicillin (50 μg/ml) and induced with 0.5 mM IPTG. The bacterial pellets were harvested ∼12 h post-induction and resuspended in lysis buffer (25 mM Tris–HCl pH 8.0, 500 mM NaCl, 0.5% glycerol, and 50 mM L-arginine, DNase 250 UL/μl, protease inhibitor cocktail, 0.002% 3-CHAPS and disrupted by sonication on ice (Omni Sonic Ruptor 400 Ultrasonic Homogenizer) using 15-second bursts at 70% amplitude, 5 cycles, with 30-second cooling intervals. The bacterial lysates were centrifuged at 16,000*g* for 20 min, and the supernatants containing the recombinant MBP-Spe3, MBP-Spe4, and MBP-PfSPDS were collected. Next, three columns containing 500 μl of amylose magnetic beads (NEB, E8021L) were equilibrated with column buffer (200 mM NaCl, 20 mM Tris–HCl, 1 mM EDTA, 1 mM DTT). The supernatants were incubated with the amylose resin for 2.5 h at 4 °C with end-to-end rotation. The columns were washed with 10 volumes of column buffer, and proteins were eluted using 10 mM maltose (M75-100; Thermo Fisher Scientific). The purity of the recombinant MBP-Spe3, MBP-Spe4, and MBP-PfSPDS was verified by SDS-PAGE and Coomassie staining. Protein concentrations were determined using a Nanodrop spectrophotometer (Biotek SynergyMX with take3 plate; TAKE3-SN).

### Fluorescence-based APT assay

The APT enzyme reaction and subsequent detection of products (spermidine and spermine) using 1,2 DAB/β-ME were performed in sequential steps. In the first step, recombinant Spe3, Spe4, and PfSPDS were used in the APT activity assay. For determining the SPDS activity of recombinant Spe3 and PfSPDS, the enzyme reactions contained either 0.1 mM or 0.5 mM dc-SAM (BOC Biosciences), 0.1 mM or 0.5 mM putrescine, 1 mM EDTA, 1 mM DTT, 1 μg bovine serum albumin (BSA), 1 μg of either Spe3 or PfSPDS, and 50 mM potassium phosphate buffer (pH 7.5), in 50 μl total volume. The reaction was incubated at 37 °C for 60 min. For determination of SPMS activity of Spe4, the enzyme reactions contained 0.1 or 0.5 mM of dc-SAM, 0.1 or 0.5 mM spermidine, 1 mM EDTA, 1 mM DTT, 1 μg BSA, 1 μg Spe4, and 50 mM potassium phosphate buffer (pH 7.5), in 50 μl total volume. The reaction was incubated at 37 °C for 60 min. For determining the SPMS activity of PfSPDS using spermidine as a substrate, the enzyme reactions were set up containing 0.75 mM dc-SAM, 0.5 mM spermidine, 1 mM EDTA, 1 mM DTT, 1 μg BSA, 1 μg Spe4, and 50 mM potassium phosphate buffer (pH 7.5), in 50 μl total volume. The reaction was incubated at 37 °C for 60 min. Following incubation, the enzyme activities were terminated by the addition of detection buffer with an alkaline pH. 35 μl of the above enzyme reactions were mixed with 85 μl detection buffer (1.75 mM β-ME, 64.5 mM sodium tetraborate buffer (pH 9.6), 0.22 mM potassium phosphate buffer, 1.48 mM 1,2 DAB) in a 96-well black clear bottom plate (265301, Thermo Fisher Scientific) and allowed to incubate at 22 °C (room temperature) for 60 min. Following this step, total fluorescence intensities (λ_ex_ = 364 nm and λ_em_ = 425 nm) were measured using a plate reader (Biotek Synergy H1, Agilent), and data was analyzed in GraphPad prism (https://www.graphpad.com/). The dual activity of PfSPDS was also tested using putrescine as a substrate. Briefly, the enzyme reactions were set up with 1 μg active or heat inactivated PfSPDS, 0.5 mM putrescine, 1 mM dc-SAM, 1 mM EDTA, 1 mM DTT, 1 μg BSA, and 50 mM potassium phosphate buffer (pH 7.5), in 50 μl total volume. The enzyme reactions were incubated at 37 °C for 90 min. The reactions were stopped by heat inactivation at 85 °C for 15 min. The reactions were precipitated with three volumes of cold acetonitrile, and the samples were subjected to LC-MS analysis to identify the substrate (putrescine) and products (spermidine and spermine).

### Polyamine analysis by TLC

Product formation from putrescine or spermidine and dcSAM substrates was confirmed by TLC on Silica 60 plates (Merck; 500 μm) using solvent system consisting of n-butanol-acetic acid-pyridine-water (3:3:2:1, v/v/v/v). Polyamines were visualized with ninhydrin spray, followed by incubation at 110 °C for 5 min.

### Analysis of polyamine-DAB adducts using FIA-MS and MS/MS fragmentation

For FIA-MS, 2 μl of each sample was injected into a Sciex 4000 QTRAP mass spectrometer operated in ESI positive mode with Q1 MS full scan data acquisition from *m/z* 50 to 2000. The gradient mobile phase was delivered at a flow rate of 0.2 ml/min, with 80% methanol and 20% water as the mobile solvents. MS/MS data were obtained using product ion MS/MS scan mode to detect fragmentation products of specified masses.

For LC-MS analysis, a binary solvent system was used, with buffer A as 100% water, 0.1% formic acid and buffer B as 100% acetonitrile, and 0.1% formic acid. Trapping was performed at 5 μl/min, 97% buffer A for 3 min using a Waters Symmetry C18 180 μm × 20 mm trap column. Samples were separated using an ACQUITY UPLC PST (BEH) C18 nanoACQUITY Column 1.7 μm, 75 μm × 250 mm (37 °C) and eluted at 300 nl/min with the following gradient: 3% buffer B at initial conditions; 5% B at 1 min; 30% B at 10 min; 50% B at 20 min; 95% B at 25 to 35 min; return to initial conditions at 40 to 55 min. MS was acquired in the Orbitrap in profile mode over the 50 to 700 *m/z* range using wide quadrapole isolation, 1 microscan, 120,000 resolution, automatic gain control target of 4E5, and a maximum injection time of 60 ms. Data-dependent MS/MS were collected in top speed mode with a 3s cycle time on species with an intensity threshold of 5E4, charge states 2 to 8, peptide monoisotopic precursor selection preferred. Dynamic exclusion was set to 30 s. MS/MS were acquired in the Orbitrap in centroid mode using quadropole isolation (window 1.6 *m/z*), high-energy collisional dissociation activation with a collision energy of 28%, 1 microscan, 60,000 resolution, automatic gain control target of 1E5, maximum injection time of 100 ms.

The concentrations of putrescine, spermidine, and spermine were measured by API 4000 QTrap mass spectrometer (Applied Biosystems Sciex) coupled with Agilent HP1200 HPLC system (Agilent Technologies). A targeted multiple reaction monitoring method was utilized to quantify the level of putrescine, spermidine, and spermine. The standards were prepared at the concentration range of 25 to 2500 ng/ml, quality controls were prepared at the concentration of 100, 500, and 2000 ng/ml. Analyst 1.7 software (https://sciex.com/products/software/analyst-software/) was used for data acquisition and analysis (Applied Biosystems Sciex). An Agilent Eclipse XDB-C18 column (3.5 micros, 2.1 × 100 mm) coupled with Agilent C18 guard column was utilized for the liquid chromatography separation at 50 °C. The gradient started with 98% of 0.1% formic acid in water (A) and 2% of 0.1% formic acid in acetonitrile (B), maintained for 0.2 min and increased to 30% B in 4.3 min, further increased to 50% B in 1.5 min and 85% in 0.5 min and maintained at 85% B for 1.5 min and went back to 2% B in 0.5 min and equilibrate for 3.5 min before next injection.

API 4000 Qtrap mass spectrometer was operated using an electrospray ionization source in positive ion mode. Multiple reaction monitoring transition monitored for putrescine, spermidine, and spermine were 89.1/72.0, 146.2/72.0, and 203.2/129.1, respectively. The declustering, potentials were 48 eV, 35 eV, and 60 eV, entrance potentials were 5 eV, 5 eV, and 9 eV, collision cell exit potentials were 10 eV each, and collision energy were 14 eV, 20 eV, and 17 eV, respectively. The ion spray voltage was 5500 eV, source temperature was 400 °C and ion source gases 1 and 2 pressures were both 50 psi, curtain gas, and collisionally activated dissociation gas was set as 15 psi and high mode.

### Data analysis to determine *Z*′ score

The S/B ratios for ySpe3 and ySpe4 were calculated using the following equation:

$$\frac{Signal}{background},\frac{S}{B}=Average\;FI\;from\;active\;enzyme/Average\;FI\;from\;inactive\;enzyme$$

FI:Fluorescentintensity

The CVs were calculated using the following formula:

Coefficientofvariation(CV)=SDofFI/AverageofFIx100

The *Z*′ factor was calculated using the following equation:

$$Z^{\prime }=1-3*\frac{\left[SD\;of\;active\;Enz+SD\;of\;inactivated\;Enz\right]}{[Fluorescence\;value\;\left(Mean\;of\;active\;Enz\right)-Fluorescence\;value\;\left(Mean\;of\;inactivated\;Enz\right)]}$$

## Data availability

All data are contained in the article and the supplemental data files.

## Supporting information

This article contains supporting information.

## Conflict of interest

C. B. M. is listed on a provisional patent application on the use of DAB-APT assay and its use to discover inhibitors of APT enzymes. The other authors declare that they have no conflicts of interest with the contents of this article.
