# Supplementary material for: A fluorescence-based assay for measuring polyamine biosynthesis aminopropyl transferase–mediated catalysis
Source: J Biol Chem. 2024 Sep 27;300(11):107832. doi: 10.1016/j.jbc.2024.107832 (PMC11541840; doi:10.1016/j.jbc.2024.107832)
Supplement: Supplemental Data — Figures S1–S10 [file mmc1.docx]

**Figure S1.** **Time-dependent and concentration dependent increase in fluorescence intensity following interaction of 1,2 DAB with polyamines. A.** Total fluorescence (λ_ex_ = 364 nm and λ_em_ = 425 nm) from 1,2 DAB/ β-ME-putrescine adducts at different concentrations of putrescine (10-100 μM) over a span of 0-160 minutes. **B**. Total fluorescence from 1,2 DAB/ β-ME-spermidine adducts at different concentrations of spermidine (10-100 μM) over a span of 0-160 minutes. **C**. Total fluorescence from 1,2 DAB/ β-ME-spermine adducts at different concentrations of spermine (10-100 μM) over a span of 0-160 minutes. **D**. Total fluorescent intensities of adducts formed between 1,2 DAB/ β-ME and 100 μM of either putrescine, spermidine, and spermine after 60 minutes of reaction. Significant difference in total fluorescent intensities were observed between PUT-SPD (p = 0.0001, PUT-SPM (p < 0.0001), and SPD-SPM (p = 0.0006). The data are presented from three independent experiments performed in triplicates, and values are mean ± S.D. The statistical significance was calculated using Welch’s t-test.

**Figure S2. A.** Putrescine-DAB LC-HRMS. **B.** Putrescine-DAB MS/MS Fragmentation.

**Figure S3. A.** Spermidine-DAB LC-HRMS. **B.** Spermidine-DAB MS/MS Fragmentation.

**Figure S4. A.** Spermine-DAB LC-HRMS. **B.** Spermine-DAB MS/MS Fragmentation.

**Figure S5. A.** Putrescine-DAB FIA-MS Full Scan. **B.** Putrescine-DAB MS/MS Fragmentation.

**Figure S6. A.** Spermidine-DAB FIA-MS Full Scan. **B.** Spermidine-DAB MS/MS Fragmentation.

**Figure S7. A.** Spermine-DAB FIA-MS Full Scan. **B.** Spermine-DAB MS/MS Fragmentation.

**Figure S8**. Purification of recombinant MBP-tagged SPE3, SPE4, PfSPDS, PfSPDS^D127A^, PfSPDS^E147A^, and PfSPDS^D127A, E147A,D196A^.

**Figure S9.** Multiple sequence alignment of *P. falciparum* spermidine synthase (PfSPDS) with spermidine synthase enzymes from *E. coli, S. cerevisiae*, and *H. sapiens*. Conserved active site residues are highlighted in red color.

**Figure S10.** Conversion rates of Spe3, Spe4, and PfSPDS enzymes. **A.** Conversion rate of putrescine, dcSAM by heat inactivated and active Spe3 is shown as a function of putrescine consumption rate based on putrescine signal intensities from TLC data in figure 4E. **B.** Conversion rate of spermidine, and dcSAM by heat inactivated and active Spe4 is shown as a function of spermidine consumption rate based on spermidine signal intensities from TLC data in figure 4E. **C.** Conversion rate of putrescine, dcSAM by heat inactivated PfSPDS, active, and triple mutant PfSPDS is shown as a function of putrescine consumption rate based on putrescine signal intensities from TLC data in figure 6D**. D.** Conversion rate of spermidine, dcSAM by heat inactivated PfSPDS, and active is shown as a function of spermidine consumption rate based on spermidine signal intensities from TLC data in figure 6E.
